# Analysis of Teaching Practices During the COVID-19 Pandemic: Teachers’ Goals and Activities in Virtual Classrooms

**DOI:** 10.3389/fpsyg.2022.870903

**Published:** 2022-04-11

**Authors:** María-Puy Pérez Echeverría, Juan-Ignacio Pozo, Beatriz Cabellos

**Affiliations:** Department of Basic Psychology, Faculty of Psychology, Autonomous University of Madrid, Madrid, Spain

**Keywords:** teaching activities, learning goals, learning outcomes, reproductive learning, constructive learning, teaching profiles

## Abstract

To research teachers’ priorities on what was to be taught and learned during the COVID-19 lockdown, we asked Spanish Primary and Secondary teachers to choose and describe the activity they preferred among those carried out with their students during the pandemic. Our interest was to investigate what really happened in the classrooms, the type of learning favored by the practices (reproductive vs. constructive), and the agreement between the teacher’s goals and their teaching We obtained 272 activities that we analyzed according to the proposed goals, the types of learning worked (verbal, procedural, and attitudinal), and the kind of teaching promoted (content or student-centered). Results showed that most teachers proposed content-centered activities, oriented above all to verbal learning. There were clear differences between the proposed goals, partly student-centered, and what was really taught, essentially content-centered. We obtained two teaching profiles, one reproductive and the other constructive.

## Introduction

The sudden closure of educational institutions in 2020, when almost 200 countries closed their classrooms because of the pandemic (ACAPS, 2020), may be considered a critical planet incident (Monereo, 2021). A critical incident is an unexpected situation which hinders the development of regular planned activity, destabilizing the system. It has clear emotional consequences, forcing normal responses to be modified and leading to a crisis of identity (Butterfield et al., 2005). This may result in profound changes provided that they are used to reflect on our theories and practices, or, in contrast, it may highlight our most deeply rooted conceptions, without promoting any change to them.

Following the impact of the COVID-19 pandemic, the countries with sufficient technological resources used Information and Communication Technologies (hereinafter ICT) to design, in a somewhat impromptu manner, a remote online and offline education. As we are reminded by Hodges et al. (2020), due to the pandemic, this remote learning cannot be regarded as planned online learning aimed at specific goals. However, as a critical incident, it may serve as a window to reveal the deeply rooted conceptions or beliefs of teachers on what and how teaching should be provided. In our case, with an extensive sample of primary and secondary education teachers in Spain (264 teachers), we wished to probe into what the teaching activity practices with their students were, and how these reflected their priorities on what was to be taught and learned during such critical times.

From that time in March 2020 and onwards, numerous reports and studies have been produced, aimed at analyzing the beliefs, experiences, and evaluations of students and teachers at different educational levels. These studies can be classified into four broad categories. Firstly, there are studies which we may call prescriptive. These state the principles that should guide the use of ICT in the classroom but fail to analyze how these activities took place actually in the classroom (see, for instance, Ferdig et al., 2020; Rapanta et al., 2020) Secondly, there are several studies which have analyzed the educational consequences of the pandemic in social aspects, particularly with regard to the increase of inequalities as a consequence of the differences in the support that the family can provide and the socio-economic context of schools (ACAPS, 2020; Andrew et al., 2020; Crawford et al., 2020; Reimers and Schleicher, 2020; Zhang et al., 2020). A third study group, more closely linked to this study, used questionnaires to probe into how students, their families, and teachers experienced virtual learning during the pandemic (Luengo and Manso, 2020; Brooks and Gierdowski, 2021) or to discover what activities the teachers considered the most appropriate (Devitt et al., 2020; Luengo and Manso, 2020; Tartavulea et al., 2020; Trujillo-Sáez et al., 2020). Lastly, a small number of studies probed into educational practices during the pandemic with previously selected samples of teachers, using case analysis (Hall et al., 2020; Iivari et al., 2020; Koçoglu and Tekdal, 2020; Rasmitadila et al., 2020).

As a whole, there are very few studies which, rather than focusing on the evaluations made by these educational agents, have researched what actually took place in the classrooms during those months. Occasionally educational priorities were questioned at a time when, due to school closure, it was necessary to readdress not just how to teach but also *what* needed teaching in that situation. Thus, for example, Trujillo-Sáez et al. (2020) found that over 60% of the teachers surveyed were more concerned about developing the competencies of their students, managing their motivation and their emotional response to the crisis, compared with the 42% who considered it a priority to ensure content acquisition in keeping with educational and developmental stages. However, compared with these priorities expressed as opinions or evaluations, other studies which researched what teachers said they had done in this situation, showed that the most common ICT use by teachers was to upload materials to a platform (Tartavulea et al., 2020) and most activities were teacher-centered (Koçoglu and Tekdal, 2020). In other words, these activities were essentially aimed at the content taught by the teachers. Therefore, in the few available studies on teaching there is a discrepancy between the goals or objectives proposed by the teachers (e.g., Trujillo-Sáez et al., 2020) and the activities which really took place (e.g., Tartavulea et al., 2020; Pozo et al., 2021).

What is the reason for this discrepancy? According to a generally accepted distinction (Ananiadou and Claro, 2009; Ertmer et al., 2015; OECD, 2019, 2020) differentiation is usually made between teacher-centered or content-centered ICT—when technologies mainly serve for the presentation of teachings or information to the students- and student-centered uses—when they are used to develop competencies in students and to help them manage their own intellectual or emotional states. This distinction emphasizes the need to replace traditional orientation based on the curricular, to the transmission of contents which increasingly prioritize the development of student competencies. This curricular reorientation has been broadly accepted in many different countries and by many international projects like, for example, the PISA report (OECD, 2019) where different types of competencies are studied. From the competency-based approach it is accepted that, although specific contents or knowledge are a necessary means of developing student competencies, the true goal of education would be to promote profound changes in students’ ability to know what to do with that knowledge and not the mere accumulation of the knowledge in itself (Claxton and Lucas, 2015; Pozo, 2016). The content-centered teachers, interested in teaching specific knowledge, also tend to maintain more reproductive conceptions and practices of learning, in which knowledge is measured by the closeness between the results obtained by the learners and the content to be taught (Bautista et al., 2009; López-Íñiguez et al., 2014). However, student-centered teachers tend to maintain conceptions of teaching and learning more based on constructive positions according to which learning outcomes are a complex and interactive construction between the conditions of the learner, the context, and the modes of teaching (Pozo et al., 2006, 2021; Pérez-Echeverría, 2022).

However, several authors emphasize that redirecting these priorities in the classroom, changing the educational goal, and conceiving of specific knowledge as a means toward that goal and not as an end in itself, requires a genuine change in mentality and teaching practices (Hong, 2012; Murphy et al., 2021; Tahirsylaj and Fazliu, 2021) or, if we prefer, a genuine conceptual change (Pozo et al., 2006; Ajayi, 2013), that is difficult to achieve. Indeed, several studies conducted prior to the pandemic found there was a gap between what teachers prefer and what they really do, as shown by the outcomes of the TALIS project (OECD, 2009; Berger et al., 2018), that is, between their conceptions and their practices. Thus, for example, Kaymakamoglu (2018) found that while teachers’ beliefs were divided between content-centered and student-centered learning, their practices were essentially traditional and centered on the transmission of knowledge to the students. In general, these studies seem to show that teachers’ conceptions are usually more complex than their practices. While their objectives readily accept that content has to be secondary to the development of abilities in their students, their practices seem to focus more on the transmission of established knowledge.

This gap between what is said and what is actually carried out reflects the plurality of representations teachers maintain (Atkinson and Claxton, 2000; Pérez-Echeverría, 2022). According to this interpretation, their explicit beliefs, what they say or believe, would be closer to constructive, student-centered approaches, or at least both types of conceptions would coexist, in an intermediate or interpretative conception (OECD, 2009; Pozo et al., 2021; Pérez-Echeverría, 2022). In contrast, their implicit conceptions, underlying their routine practices in the classroom, would tend toward traditional knowledge transmission. This would at least partially explain this discrepancy between what teachers say (their beliefs) and what they do (their practices; Pozo et al., 2010; Buehl and Beck, 2015). Their practices are not totally homogenous either. Although one type of practice predominates over others, teachers usually use a variety of them (Vieluf et al., 2012; Pozo et al., 2021). Thus, the critical incident that closed down schools as a consequence of the COVID-19 pandemic was an excellent opportunity to unveil these implicit conceptions, insofar as, faced with these incidents, a common response would be to use regular routines or practices, that provide us with the certainty of what we already know, in times of anxiety or uncertainty (Vandercleyen et al., 2014).

Therefore, in this study, we have proposed to research whether the activities carried out by teachers through ICT during lockdown education were aimed at fostering the development of students’ competencies or were solely used for the transmission of knowledge or educational contents. However, it was also of interest to investigate what is being taught and whether this teaching is content or competency-aimed because they reflect different conceptions and priorities about the curriculum. Different categorizations of learning outcomes have been proposed, most of them based on the classic taxonomy of Bloom (1956). For example, Nusche (2008) differentiates between cognitive outcomes (knowledge and skills) and non-cognitive outcomes (attitudes, values, and emotions). For his part Claxton (2018, 2021) distinguishes three “layers of the river of learning”: knowledge and comprehension (more superficial, easy to observe, and flowing), skills and literacies (these move underneath the former, and flow is slower and more difficult to perceive), and attitudes and dispositions, which move around at the deepest part of the rive of learning and usually lead to slower, more gradual changes which are also initially less visible. Using a similar distinction, in a previous study also conducted during the COVID-19 pandemic, we asked the teachers to inform us of the frequency with which they set out to do different activities (Pozo et al., 2021). These activities showed different types of teaching goals and outcomes: verbal knowledge, procedural and attitudinal and, within them, more or less directed toward knowledge, skills, or values in Claxton’s words Claxton (2018, 2021). We will also use this distinction in the analysis of activities in this work. This confirmed that on all educational levels and areas studied teaching activities mainly aimed at the transmission of verbal knowledge and sticking to routines and attitudes toward learning rather than the learning of skills or values or attitudes. Equally, in that same study, we confirmed that most of these activities aimed to promote reproductive learning, centered on the accumulation of knowledge instead of the development of students’ competencies.

Notwithstanding, most studies conducted on teaching and learning developed during COVID asked teachers (Devitt et al., 2020; Rasmitadila et al., 2020) or students (Ardini et al., 2020) to undertake an evaluation of their preferences, or, at best, recognize the type of practices they had made (Pozo et al., 2021). However, as we have already pointed out, there are many studies that flag up the gap between what the teachers say and what they do (Korthagen, 2010; Pozo et al., 2010; Buehl and Beck, 2015; Clarà, 2019; Pérez-Echeverría, 2022). In the case of ICT usage, we observed, for example, that the teachers said they preferred student-centered uses, but finally they admitted that in practice they mainly used them to transmit information and knowledge to their students (de Aldama and Pozo, 2016).

As we said previously, several studies had probed into teaching practices during the pandemic using case analysis, made with small and selected samples (Aznar-Sala, 2020; Basilaia and Kvavadze, 2020) that may not be representative of all teachers. To access a larger and more representative sample, it is necessary to carry out other types of tasks based on a non-observational methodology. For this reason, just as de Aldama and Pozo (2016) had done, we asked the teachers to describe to us the activities they had done in the virtual classrooms. Although with this methodology we were unable to analyze real practices, which is only viable with very small samples, it did provide us with information on the practices declared by the teachers and we could reach a more representative sample. We were also able to contrast the effect of different variables on these practices, on the assumption that there were different types of teachers and that some of these differences may be linked to variables that could predict them, such as gender, years of experience, level, or material taught. However, in many cases, the data obtained on the effect of these variables was not conclusive. Thus, for example, in relation to the impact of gender on teachers, while in some studies, women were shown to have a closer student-centered approach than men (Norton et al., 2005; Laird et al., 2011) in others they were shown to be more traditional than the men (Fan and Ye, 2007) or in some that there were no differences in relation to gender (Stes et al., 2008). Ambiguous data also emerge regarding the impact of teaching experience. In some studies, it was found that the most expert teachers (or those who were older, since these two variables are hardly separable) displayed more traditional conceptions than the most inexperienced teachers (and also the youngest; e.g., Bautista, et al., 2009; López-Íñiguez et al., 2014), whereas other studies found that the conceptions and practices of the most experienced teachers were more student-centered (Fives and Buehl, 2010; Rubie-Davies et al., 2012; Pozo et al., 2021) and still others found there were no conclusive differences in teaching practices (Norton et al., 2005; Graham et al., 2020).

Concerning the educational stage and material taught, some studies show that the more advanced the stage and higher the theoretical density of what is being taught, the conceptions are more content or teacher-centered (Martín et al., 2014), a data confirmed by Pozo et al. (2021). Other authors did not find there was any difference with regard to this variable (e.g., Weiqiao and Shengquan, 2007). For their part, in a study with university professors, Laird et al. (2011) found that in the “toughest” subjects (such as science and mathematics) a teacher-centered approach to education was more common. Moreover, it has consistently been found that the previous use of ICT in the classroom not only promotes greater willingness to use these technologies but also fosters more student-centered conceptions and practices (Inan and Lowther, 2010; Pozo et al., 2021).

Possibly one of the reasons why the impact of these different variables is so unstable is that the teachers do not have a single conception or idea of what is happening in the classroom but have a number of ideas which they update according to the context (Korthagen, 2010; Clarà, 2019). These different ideas are not in disarray; however, they are grouped around characteristic profiles (Bautista et al., 2009; Vieluf et al., 2012; Jacobs et al., 2014; López-Iniguez et al., 2014; Pérez-Echeverría, 2022). In our previous study (Pozo et al., 2021) we found four teaching profiles which varied according to the amount of work carried out with students during the pandemic and the direction of these practices, more or less centered on the reproduction or construction of knowledge, or as previously stated, on the contents or the students. Out of these four profiles, only one represented teachers who said they carried out constructive practices together with other more reproductive ones. For their part, Vieluf et al. (2012), in a reanalysis of data from the TALIS (OECD, 2009) report, with over 70,000 teachers from 23 countries, it was found that there were three teacher profiles: one which highly frequently used structuring, student orientation and enhanced teaching practices and pedagogical innovation; another group which used these practices much less frequently, and a third which was positioned in-between these two groups.

For all of these reasons, as shown in our objectives below, when analyzing the types of activities teachers planned during the pandemic as a means of accessing their educational priorities, we will consider to what extent they are influenced by different teaching variables (gender, years of experience, educational level and material taught, prior use of ICT, etc.) so as to also try to identify different teaching profiles regarding the goals and types of learning involved in education.

From the reflections and priorities established in the Introduction we proposed the following objectives:

1. Identify teachers’ educational priorities during lockdown education, from the goals initially proposed and the activities which were actually carried out.

1.1 Analyze the type of goals proposed according to outcome sought (verbal, procedural, or attitudinal) and the learning promoted (reproductive or constructive).

1.2 Analyze the contents worked on in the classroom, bearing in mind these two same dimensions, the outcomes of learning, and the type of learning promoted.
2. Identify whether the activities carried out really promote the proposed learning.

2.1 Analyze the relationship between proposed goals and practices carried out in the classroom from the viewpoint of whether they were centered as reproductive (content or teacher-centered) or constructive (student-centered).
2.2 Analyze whether the activities done were aimed at teaching content (content-centered) or the formation of competencies (student-centered) and, in this case, what type of competencies they were aimed at (area/digital/transversal).

3. Ascertain which variables impact these priorities with respect to what is taught (gender, experience, level, area, previous use of resources, digital resources used).
4. Identify possible teaching profiles or styles in the activities carried out and relate them to the previous mentioned variables.

## Materials and Methods

### Task and Procedure

In the framework of a broader research study, between May and June 2020, while schools stayed closed due to COVID-19, primary and secondary teachers in Spain were asked to choose an activity they had done during the pandemic that was representative of his work. Teachers were to describe their activities in their own words, to the extent they deemed necessary. They were instructed to include in this description the objectives they set, the results they hoped to achieve, the activities to be carried out by the students, and the forms of evaluation. It was therefore an open task. The Qualtrics tool was used to introduce the task and was sent to teachers throughout Spain. To foster participation, a 75€ raffle in educational material was used.

### Participants

We used directories of emails from public, private schools, and high schools of Spain to get in contact with the participants. After completing a questionnaire used in a previous study, which included the demographic data of the participants (Pozo et al., 2021), the teachers were asked to describe an activity they had done with their students in the virtual classroom. Of the 1,403 teachers who had completed the questionnaire, 287 sent a description of an activity. Six of these teachers sent in two activities and one sent three. We eliminated from the sample the activities of 23 teachers which did not contain sufficient information for analysis. All activities that did not describe the objectives or tasks that students had to perform were eliminated since in those cases it was not possible to categorize the activities. We, therefore, studied 272 activities. The personal and professional variables of the participants (gender; age; years of teaching experience; stage and specialty of classes and previous ICT experience).

### Design

We used an *ex post facto* retrospective design. The independent variables were participant teacher characteristics (gender; age; years of teaching experience; stage and specialty of classes; previous ICT usage and digital resources of the students), (see Table 1).

**Table 1:** Participant characteristics.

Variable	Categories	Category N ^*^
Gender	Men	77
	Women	193
	Non-binary^**^	2
Teaching experience	5 years or fewer	80
	From 6 to 15 years	73
	From 16 to 25 years	76
	26 years or more	43
Educational level	1°, 2° o 3° Stage of Primary Education (6–9 years)	62
	4°, 5° o 6° of Primary Education (9–12 years)	69
	Compulsory Secondary Education (12–16 years)	114
	Non-compulsory Secondary Education (12–18 years)	27
Primary Curriculum Subjects	Generalists	69
	Specialists	58
Secondary Curriculum Subjects	Spanish language	25
	Mathematics	12
	Social Sciences	15
	Natural Sciences	26
	Foreign Language	19
	Technology	14
	Others^***^	29
Previous ICT use	Never	64
	Someday per month	126
	Someday per week	49
	Everyday	33
Digital resources	Under half	16
	Around half	31
	Most	166
	All	59

* *The number of participants may vary slightly from one variable to another due to some teachers not providing information on that variable*.

** *In gender analysis non-binary participants were not considered because it was a very small sample*.

*** *The category “others” referred to specialties with a very small number of teachers*.

The dependent study variables were the activities reported by teachers, analyzed in keeping with a category system. For this categorization, we adapted the System of Analysis of Practices of Instruction and Learning (SAPIL; Pozo et al., 2022) which had been used in previous studies, especially for the observation of music education activities (López-Iniguez and Pozo, 2016; Casas-Mas et al., 2019) but also in other areas (de Aldama and Pozo, 2016). This system distinguishes three components: what is taught (outcomes or contents); the student’s mental activity to achieve this learning (Processes), and the educational practices designed by the teacher to do so (Conditions). For this study, according to the previously mentioned objectives, we developed from SAPIL a new system that we call SATA (System of Analysis of Teachers Activities), establishing differentiated categories for (a) initial goals explicitly proposed by the teacher for the activities and (b) activities which were really used. Both with respect to the proposed goals and the contents used in the classroom, the system also distinguished between reproductive (teacher or content-centered) and constructive (student-centered) learning, and also between those aimed at verbal, procedural, or attitudinal outcomes. We thus differentiated between activities more aimed at competencies and those where the objective was solely knowledge acquisition. Competencies were divided into those in keeping with the area, transversal (learning to learn, critical thinking, etc.) or digital. The Appendix contains the definitions of each category and examples extracted from the answers of the teachers to fine-tune the SATA design, we performed an interjudge analysis both for the final definition of the categories and for inclusion of the activities in them. During phase one, the judges worked on the selection of categories and the examination of some activities. Once an initial analysis system had been performed, during the second phase, two groups of three judges, who were collaborators on the team of research, analyzed 50 activities (17.40% of the total), randomly chosen, obtaining a high interjudge agreement (Kappa indexes of 0.782 and 0.858). This served to remove defined categories which were not used in the activities and to adjust the inclusion criteria in the different categories, thereby adjusting their definition. After this analysis, the other activities were randomly divided between the two groups. Each one of the groups of activities was independently analyzed by two judges, from the previous team. Category inclusion disparities were resolved through discussion and consensus.

### Data Analysis

Since some teachers reported on more than one activity, the analyses were performed bearing in mind all the activities presented, and therefore the total number did not coincide with the number of teachers.

To study objectives 1 and 2—types of goal (verbal, procedural, attitudinal; reproductive, constructive) and types of outcomes proposed (verbal, procedural attitudinal; reproductive, constructive), the frequencies of each category were counted and the differences between them were analyzed using Cronbach’s Q and McNemar’s test statistics to identify where the differences between goals and outcomes or between different types of outcomes occurred. Cronbach’s Q and McNemar’s test statistics were also used to analyze the differences between the different competencies described and a binary logistic regression was performed to identify which competencies better explained the competency activity carried out.

Data relating to objective 3 (influence of the different independent variables in the types of goals and types of outcomes) were analyzed using chi-square and their Adjusted Standardized Residuals (ASR), taking as independent variables the personal and professional characteristics. The relationship between these independent variables and the profiles relating to objective 4 were also analyzed.

To study objective 4 which was to identify possible teaching styles, a hierarchical type of cluster analyses was used, made from the categories. All statistical analyses were performed using SPPS version 26.

## Results

We will present our results in the same order as that followed for the objectives, except in the case of objective 3, which refers to the impact of the different independent variables, the effect of which we will describe in relation to each of the other three objectives. We will therefore begin by analyzing the goals proposed by teachers according to the type of learning promoted (objective 1.1) and we will then study the types of outcomes studied in the activities (objective 1.2).

**Figure 1:**
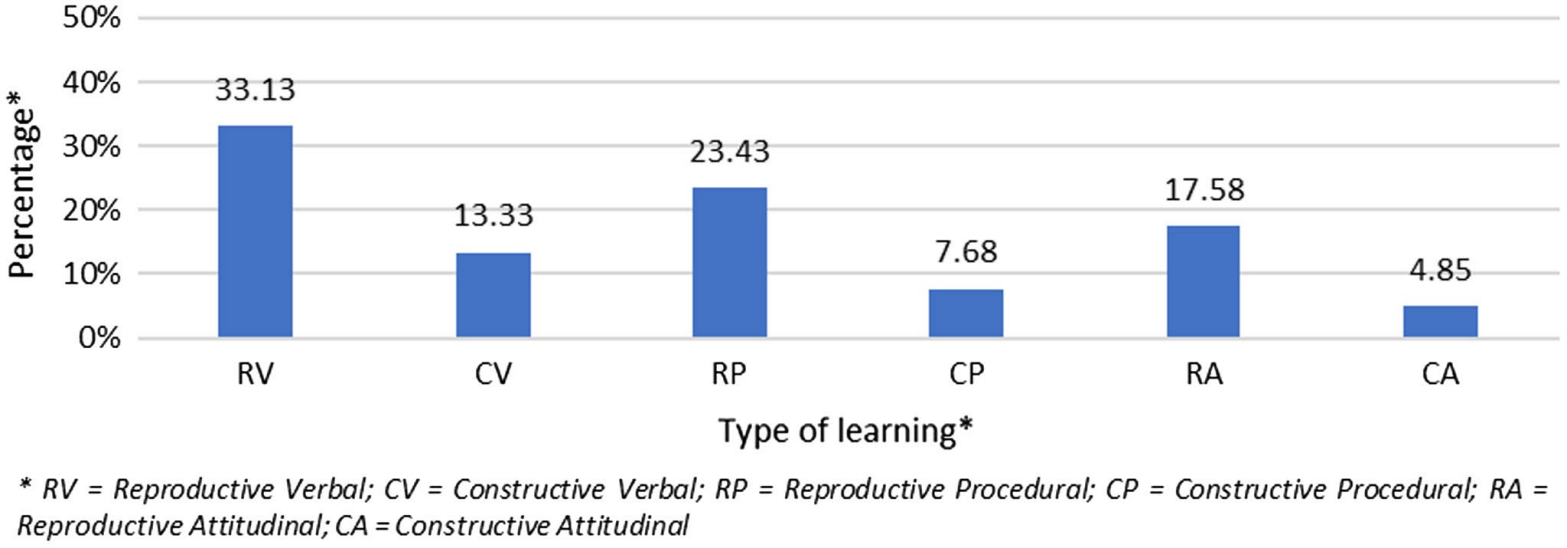
Percentage of goals proposed by the teachers arranged according to type of learning.

As may be seen in Figure 1, the activities are aimed mainly at reproductive goals or more content-centered than student-centered (Cochran’s Q = 111.74; *p <* 0.001). In fact, 74.10% of the proposed activities have reproductive goals. Only a quarter of the goals proposed by teachers for their activities are constructive (25.80%), or student-centered. There are also significant differences between goals aimed at verbal (46.46%), procedural (31.11%) and attitudinal (22.42%) learning (Cochran’s Q = 47.87; *p <* 0.001).

**Figure 2:**
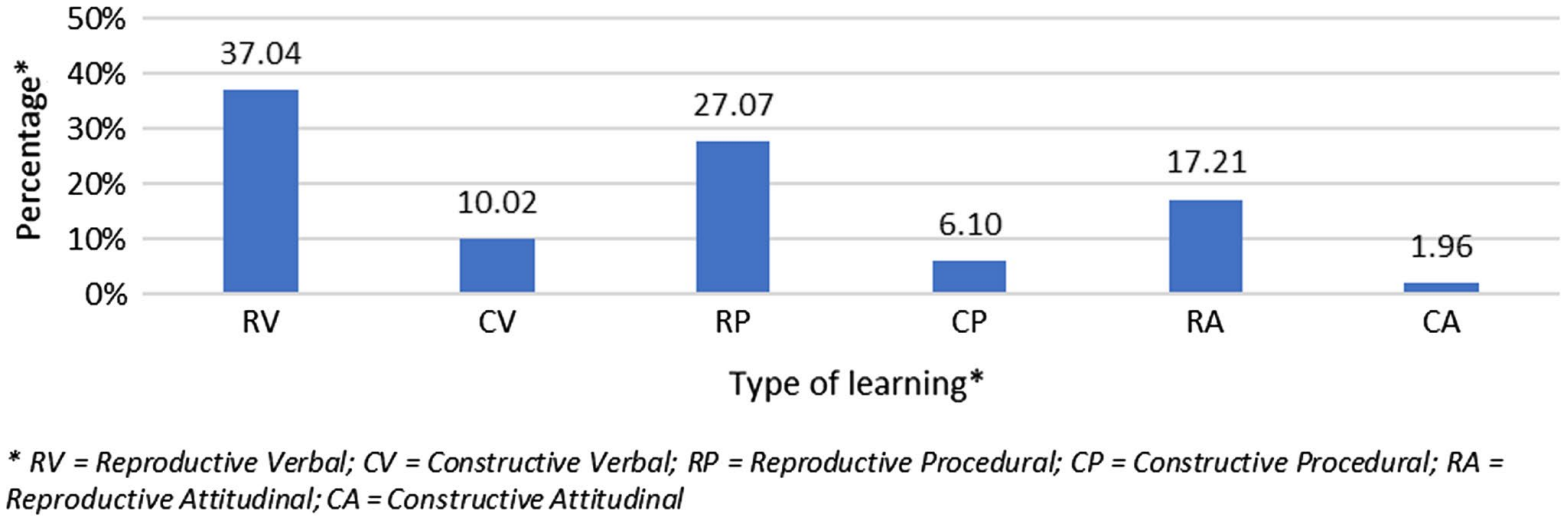
Percentage of types of learning involved in the activities.

These same differences are repeated and even increase when we analyze the learning outcomes that have really been worked on (objective 1.2; see Figure 2). 81.92% of the activities that teachers plan for their students are reproductive or content-centered, compared with 18.08% which are student-centered or constructive (*Q* = 158.72; *p <* 0.001). Almost half of these activities (47.06%) aim at obtaining verbal outcomes, 33.77% work on procedures, and 19.17% on attitudes (*Q* = 67.90; *p <* 0.001).

We were also interested in comparing the proposed goals with the activities actually carried out (objective 2.1.). McNemar’s statistics showed that there were no differences between the goals aimed at reproductive learning and the reproductive learning involved in the activities. However, the constructive goals are more common than the activities really designed for this (*p* < 0.001). In the case of verbal learning, which is the most common, there were no differences between what was proposed and what was done but differences were found in procedural (*p* < 0.001) and attitudinal (*p* < 0.05) learning proposed and carried out. In both cases, goals aimed at these types of learning were proposed more than were really worked on. As a result, data showed differences between goals proposed and content really worked on. In general, what was proposed is more student-centered and contains more varied activities than what is really done, which centers more on the presentation by the teacher of essentially verbal learning.

Concerning objective 3, on the impact of the different variables in the learning outcomes worked on, the chi-square analysis showed that women did more reproductive activities than men (*χ*^2^ **=** 4.37, *p <* 0.05, ASR = 2.1). Regarding teaching experience, the constructive student-centered activities were most frequent in teachers with an experience between 6 and 15 years, (*χ*^2^ = 12.37, *p <* 0.01, ASR = 2.8), whereas those with teaching experience between 16 and 25 years were less student-centered (*χ*^2^ = 12.37, *p <* 0.01, ASR = 3). Neither gender, nor experience, nor type of center was related to the type of learning taking place in the classroom. However, secondary teachers proposed more procedural activities in the classroom than those of primary (*χ*^2^ = 9.872, *p <* 0.05, ASR = 2.9). For their part, according to the Fisher analysis, the teachers who proposed more activities aimed at verbal learning were the secondary teachers who taught a foreign language (*p <* 0.05, ASR = 2.2) and social sciences (*p <* 0.05, ASR = 2.2). In contrast, technology teachers did not use these verbal leanings as much (*p <* 0.05, ASR = −2.1). Here, the only different between the proposed goals was that secondary teachers proposed more constructive goals (*χ*^2^ **=** 9.4, *p < 0*.05, ASR = 2.6) than the first cycle primary teachers (*χ*^2^ = 9.44, *p <* 0.05, ASR = −2.5). To sum up, the independent variables studied were of little importance, both in goals proposed and in learning outcomes worked on, maybe because most of the activities were reproductive and therefore the general pattern was fairly homogenous.

With respect to the objective 2.2 which focuses on whether activities are aimed at competencies or the transmission of specific contents, McNemar’s analysis did not flag up any differences between the proposals aimed at competencies (44.51%), or those aimed only at content learning (55.48%). Most of the declared competencies were those in keeping with the area (59.34%) while digital competencies (20%) and transversal competencies (19.67%; *Q* = 150.2, *p* < 0.001) were less frequent. As explained in method, we used binary logistic regression analysis to confirm the relationship between the competencies which teachers stated they used and the activities they proposed to develop them. The type of competency proposed explains to 32.30% (R^2^_de Nagelkerke_ = 0.32) the competency activities carried out. The highest relationship came about between the transversal competencies, which were the least frequent, and the activities aimed at achieving them (*B* = 2.17, Wald = 37.06, *p <* 0.001). While the weakest relationship came about between digital competencies (*B* = 1.11, Wald = 10.41, *p* < 0.01) and area (*B* = 1.54, Wald = 15.49, *p <* 0.001) and the corresponding activities. In other words, the few teachers who indicated that their activities were aimed at developing transversal competencies (critical thinking, learning to learn, etc.) carried out proportionately more competency activities than those which showed area or digital competencies.

Regarding the impact of other variables (objective 3), digital competencies were more frequently proposed by secondary teachers (*χ*^2^ = 20.634, *p <* 0.001 ASR = 4) and particularly by those of technology (*p <* 0.05 ASR = 2.7), while they did not form part of the goals of the teachers of the youngest children aged between 6 and 9 years (*χ*^2^ = 20.63, *p <* 0.001). However, the area competencies, which were the most common, were not affected by any of these variables, except by the prior frequency of ICT use by the teachers. Those who used ICT prior to the pandemic included these competencies more often in their proposals (*χ*^2^ = 32.57, *p <* 0.001, ASR = 5.6), than those who had not used them (*χ*^2^ = 32.566, *p <* 0.001, ASR = −2.2). Lastly, women proposed more competencies than men (*χ*^2^ = 4.957, *p <* 0.05, ASR = 2.2). However, the frequency of activities oriented to competencies was higher in secondary school teachers than in other teachers, (*χ*^2^ = 8.67, *p <* 0.05, ASR = 2.6), while it was lower in first primary cycle teachers (*χ*^2^ = 8.668, *p < 0*.05, ASR = −2.4).

**Figure 3:**
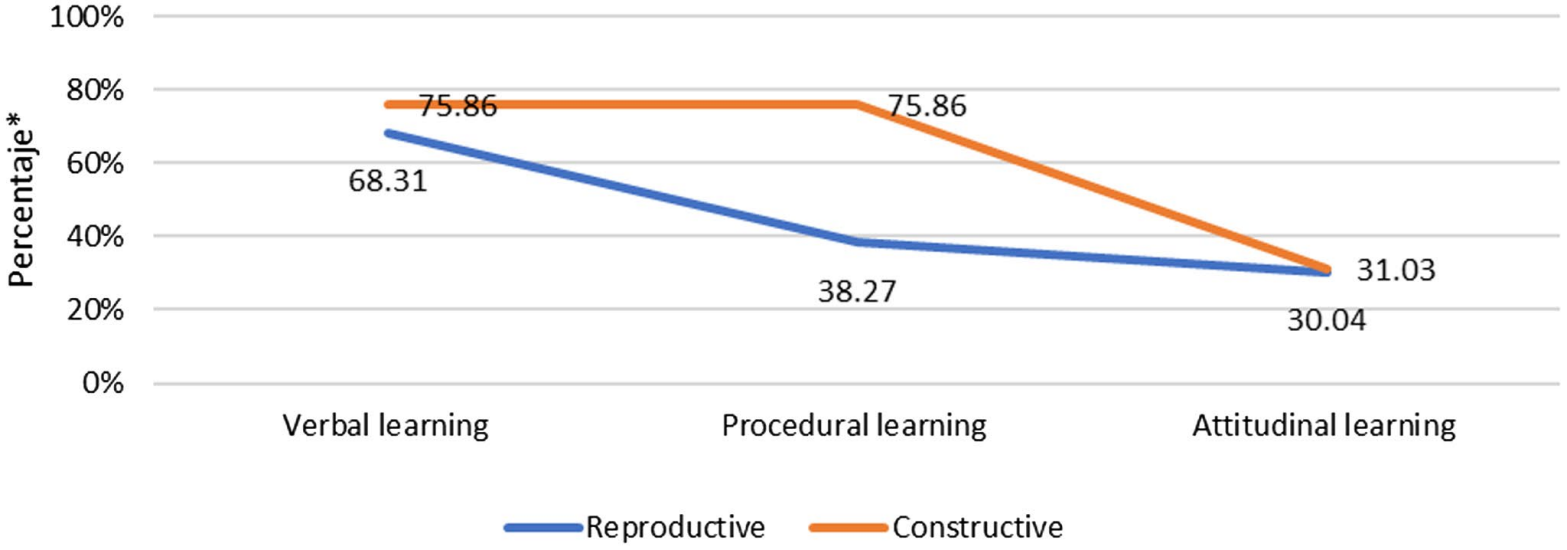
Frequency of each learning worked on in each profile.

The objective 4 was to study the possible teaching profiles or styles. After carrying out the corresponding cluster analysis, we were able to find two teaching profiles or styles (see Figure 3). One of the profiles, the most numerous, included 89.3% of the participants. This style was eminently reproductive with more verbal than procedural or attitudinal activities being carried out. Primary teachers are over-represented in this profile and no other differences were observed in relation to the other independent variables we analyzed.

The second profile included more constructive teachers which only amounted to 10.7% of participants. These teachers, unlike the reproductive cluster, centered on both verbal and procedural learning goals, but, as in the reproductive profile, they did not take into account attitudinal aspects. They mainly sought the development of digital competencies but were also oriented toward area and transversal competencies. Secondary education teachers are more associated with this profile and there are no differences regarding gender, specialty, or previous ICT usage.

To sum up, most teachers (almost 90%) fit into a “traditional” profile, aimed at reproductive and essentially verbal learning. Only 10% of these teachers propose student-centered learning, which promote constructive learning and they attach greater importance to procedural and digital competency learning. The variables studied bear little relationship to these profiles. It is only of note that secondary teachers more frequently fall into the small constructive profile group, although the great majority of these teachers also have a traditional profile.

## Discussion and Conclusion

We began this paper by considering that the closure of educational centers due to the pandemic was a global critical incident (Butterfield et al., 2005; Monereo, 2021), and we asked ourselves how this incident had been resolved by different teachers, thereby employing an analysis of the activities described by 287 primary and secondary school teachers in Spain. We wondered whether during those months the activities suggested by the teachers would be more student-centered, aiming at developing student competencies through complex, constructive learning or through teacher and content-centered learning aimed at the transmission of knowledge which the students then had to essentially learn through repetition and reproduction.

After analyzing the goals proposed by teachers and the types of content they work on in their activities (objective 1), we confirmed that both cases were essentially aimed at verbal or procedural type reproductive learning. According to these results, the teachers were hardly concerned by attitudes, and neither did they foster constructive student-centered learning. Although in our previous study with a much broader sample but with different methodology, a predominance of reproductive learning was also found (Pozo et al., 2021), in this study where teachers were asked to describe one of the activities which really took place, orientation toward reproductive learning was even more extreme. This was apparent both from the greater frequency of teacher-centered activities and because they also showed lower priority toward attitudinal learning of their students during the pandemic.

The predominance of content-centered activities aimed at reproductive learning is the main outcome of this research, from which all other outcomes should be interpreted. In fact, the priority of reproductive over constructive goals and learning is also reflected in the teacher profiles and styles (objective 4), obtained using cluster analysis, in which 90% of teachers are associated with a reproductive profile, while only 10% have a constructive profile. If by traditional education we mean that which is content-centered or which seeks reproductive learning then we may conclude that the teachers resolved the critical incident by resorting to traditional conceptions and practices (Vandercleyen et al., 2014). Although we are unaware of the activities that these teachers carried out prior to the pandemic, our data suggest that digital techniques helped the teachers to carry out teacher or content-centered practices which, according to data from before the pandemic, continue being the most common (Sigalés et al., 2008).

Notwithstanding, despite this predominance of reproductive learning, we found there were some differences between the goals and contents worked on in the virtual classrooms but there were no significant differences between the goals and what was taught when learning was reproductive. Like Koçoglu and Tekdal (2020), we observed that the teachers often propose constructive goals which in the classroom become reproductive learning activities. Again, there was therefore distance between the goals of the teachers and the practices carried out (Tartavulea et al., 2020; Pozo et al., 2021) or between the teachers’ conceptions and their practices (Berger et al., 2018; Kaymakamoglu, 2018) or between their verbal declarations and their teaching (Trujillo-Sáez et al., 2020). In this sense, it is particularly striking that in contrast to the concern shown by teachers regarding the emotional and affective problems of their students coming from lockdown and isolation during the strictest times of the pandemic (Trujillo-Sáez et al., 2020), only one-fifth of the teachers in this study (22.42%) proposed attitudinal goals in their activities and even fewer (19.17%) prioritized these learning outcomes in their classrooms.

This distance between theory and practice, between what teachers say and what they do, was again observed in the differences between the competencies the teachers wished to achieve in their students and those they really worked on (objective 2). A little under half of the teachers stated they wished to develop competencies. However, in these teachers, these competencies were only related to a third of the activities they performed. Of these competencies, the most repeatedly mentioned were those relating to the subject matter they taught, and therefore closer to contents, while the least mentioned were transversal. However, the latter were relatively more commonly proposed by teachers with a constructive profile, and in turn, were the ones which related most to competency activity.

The different independent variables related to the demographic characteristics of the participants had very little in common with the results found. Data, therefore, appear to show a homogenous pattern where the activities, regardless of these variables, were aimed at reproductive activities and outcomes.

To sum up, this study shows that teachers’ activities during the pandemic were essentially reproductive and centered more on verbal and procedural development rather than attitudinal. In this sense, a clear difference is shown in studies where teachers were asked about what their preferred activities were (for example, Ferdig et al., 2020; Rapanta et al., 2020; Sangrà et al., 2020; Zubillaga and Gortazar, 2020) and with many of the case studies based on interviews with teachers (for example, Hall et al., 2020; Iivari et al., 2020; Rasmitadila et al., 2020). In all of them, the teachers demonstrated greater proximity to constructive conceptions.

As previously indicated in the introduction, we believe these differences could be due to research methods of enquiry. If we accept the idea that teachers have many representations on learning and teaching (Atkinson and Claxton, 2000; Ertmer et al., 2015; Pérez-Echeverría, 2022) and they propose activities and carry out practices of different characteristics (Vieluf et al., 2012), we may expect that different methods of enquiry facilitate access to different representations or practices. Following this logic, we could say that our study has two clear limitations. On the one hand, we only examined one of the activities the teachers stated they had done. On the other, we did not observe their real practice. In both cases, this limitation was related to the objective of accessing a larger and more representative sample than an observational methodology would have allowed us. However, also following the logic of our argument, it could be expected that direct observation of activities would demonstrate an even more reproductive profile, although by analyzing more activities it is possible there could be more intermediate profiles.

We are aware that this method did not address the complexity of teaching work and the multiple factors involved in it. Therefore, since demographic variables were not relevant for explaining or fine-tuning the interpretations of our data, we believe that in upcoming studies further probing should be made into these variables, both regarding activity goals and learning outcomes, together with other aspects of the same (teaching organization, evaluation methods etc.). Our findings may have been the consequence of having analyzed just one activity and the presence of further activities could have led to the appearance of intermediate profiles between the two.

However, despite these limitations, the results we have found are similar to those found in other works on teaching carried out in face-to-face contexts before the COVID-19 pandemic (OECD, 2009; Berger et al., 2018), (Sigalés et al., 2008; Kaymakamoglu, 2018) so it gives the impression that the use of online resources by themselves, without other deeper changes, contributes very little to change the forms of teaching. The critical incident caused by the pandemic has brought to light the beliefs of teachers about what should be the goals of their teaching and the activities to carry them out, reflecting even a conception focused on the contents that will undoubtedly hinder a full integration of ICT in the classroom. (Ertmer et al., 2015). Given that the crisis we have experienced has undoubtedly accelerated the need to adopt hybrid approaches, which integrate the virtual and the face-to-face, lessons (Sangrà et al., 2020; Li et al., 2021; Singh et al., 2021), it seems increasingly urgent to develop teacher training programs in digital literacy (Sánchez-Cruzado et al., 2021) that not only make possible the mastery of these resources but help to rethink their role in the new educational spaces to move toward student-centered teaching, for which ICT should play an essential mediating function.

## Data Availability Statement

The raw data supporting the conclusions of this article will be made available by the authors, without undue reservation.

## Ethics Statement

The studies involving human participants were reviewed and approved by Ethics Committee of the Autonomous University of Madrid. The participants provided their written informed consent to participate in this study.

## Author Contributions

M-PE: funding acquisition, conceptualization, methodology, supervision, validation, writing – original draft, and writing – review and editing. J-IP: funding acquisition, project administration, conceptualization, methodology, supervision, writing – original draft, and writing – review and editing. BC: conceptualization, methodology, data curation, formal analysis, investigation, software, writing – original draft, writing – review and editing, and visualization. All authors contributed to the article and approved the submitted version.

## Funding

This work was supported by the Ministry of Science and Innovation of Spain (PID2020-114177RB-I00).

## Conflict of Interest

The authors declare that the research was conducted in the absence of any commercial or financial relationships that could be construed as a potential conflict of interest.

## Publisher’s Note

All claims expressed in this article are solely those of the authors and do not necessarily represent those of their affiliated organizations, or those of the publisher, the editors and the reviewers. Any product that may be evaluated in this article, or claim that may be made by its manufacturer, is not guaranteed or endorsed by the publisher.

## Figures and Tables

**Table:** 

**Category**			**Definition**	**Examples extracted from the participant descriptions**
Goals proposed	Reproductive Verbal (RV)		Those activities the teacher proposes so that their students learn facts and data.	Learn the basic characteristics of the periodic table and its organization.
	Constructive Verbal (CV)		Those activities that the teacher proposes so that their students acquire concepts through relationship and reflection between their knowledge and the new information.	It was intended that they reflect, forming their own criteria, on the links between democracy and economic and social structures or the differences between the current representative democratic model and other ways of thinking about democracy, such as the one that can be seen in Rousseau’s social contract.
	Reproductive Procedural (RP)		Those activities the teacher proposes so that their students learn routine sequences of actions or techniques.	Identify the coordinates of points from the graph, calculate the slope of a straight line from two points by counting “squares,” calculate the slope with its formula, operate with whole numbers and fractions, understand the meaning of their value and sign.
	Constructive Procedural (CP)		Those activities the teacher proposes so that their students apply self-regulated sequences of actions, that is, planning them, making decisions, and/ or controlling their application.	In the case of the baccalaureate students, the idea was to learn to pick out the main information from a long text. They were to learn how to select what to learn and how to do it most efficiently.
	Reproductive Attitudinal (RA)		Those activities the teacher proposes so that their students learn and apply self-regulated sequences of actions, that is, planning them, taking decisions and/or controlling their application.	The main objective of the activity was to interact with the family playfully and reduce tensions in the hardest days of confinement.
	Constructive Attitudinal (CA)		Those activities proposed by the teacher aimed at getting the student to reach their own moral criteria from reflection on behavior and norms or through reflecting on their own emotions and those of others, to become aware of them and/ or control them.	It was proposed that they learn, reflect, and show solidarity with the current situation caused by COVID-19. It was carried out through photographs that reflect the historical moment.
Learning Outcomes	Reproductive Verbal (RV)		Those activities where the result of learning would be the acquisition of facts and data	To watch an Edpuzzle video story and answer questions on a paper sheet that they then had to hand in.
	Constructive Verbal (CV)		Those activities where the result of learning is the acquisition of concepts or theories, that is, verbal results that acquire significance due to their relationship with other concepts or theories.	To select a work of art and its author. To describe it according to the criteria of color, composition seen in plastic and visual arts lessons and make a reflection on the time in which it was created, the political, economic, and social situation.
	Reproductive Procedural (RP)		Those activities whose result is the learning of fixed sequences of action or techniques.	Take a picture of the notebook and rename the file “homework of such and such person _1A.jpg.” Attach the picture to the email. Send it to your teacher and copy (CC) my address. In “subject” write something specific. “English homework. Such and such a person (1 A).” Write the email including greeting, polite sentence, clear and concise message, farewell, and signature (giving them an example). Check the spelling. Optionally, I suggest converting the image to PDF format.
	Constructive Procedural (CP)		Those activities promote learning results related to the acquisition of procedures adapted to the task through planning, decision-making, and application control.	Organize into groups of 5 or 6, divide the tasks, look for and summarize information on a chosen theme (the food web, the Nazis and the holocaust, coronavirus, and addictions), outline the information using PowerPoint.
	Reproductive Attitudinal (RA)		Those activities promote the acquisition of efficient responses to facilitate the student’s functional behavior or contribute to the expression of feelings and emotions.	The main objective was playful, nothing more than playful, and that they would do a bit of exercise but above that all they would have a good time, learn to interpret the song, and eliminate stage fright of individual interpretation.
	Constructive Attitudinal (CA)		Those activities promote the student reach their own moral criteria from which to reflect on the behaviors and norms, or the student reflects on their own emotions or feelings and those of other people.	For 15 days, they had to draw, illustrate, or capture, in A5 format, a feeling so that they would learn to express their feelings, in addition to experimenting with the different artistic techniques.
Competencies	Type	Area	It includes the objectives involved in the curriculum of specific areas.	To make a video tutorial of how they would determine an unknown variable in a real cinematic case made in their homes (Natural Sciences Teacher).		
		Digital	It includes those objectives aimed at initiating digital resources or their pragmatic use and the management of knowledge through digital means or their epistemic use.	To work with the tools offered by Google Gmail using the mail center: classroom, text documents, spreadsheets, presentations, meet meetings../ Management of social networks: use of hashtags, mentions, retweeting, rules of courtesy, use of social networks as a method of cultural dissemination, not only as a place for exhibiting.		
		Transversal	It includes those objectives aimed at learning skills, knowledge, and attitudes acquisition that promote better performance in different disciplines.	In groups of 4, after group cohesion dynamics, work with normal roles (moderator, spokesperson,) to learn to collaborate.	
	Competency activity		It includes those activities aimed at the achievement of competencies. This involves: -Integrating cognitive and emotional elements and different types of content Functionality Transference -Autonomous usage.	The second part of the activity consisted of in that the student recorded a video, showing a primary, secondary, or tertiary sector activity in their village (from a farm to a shop). Then they explained what activity consisted of and how it related to the subject we had been learning about in class.

## Appendix

Appendix: Categories used in the analysis of activities and examples of the categories.
